# Frailty Factors and Outcomes in Patients Undergoing Orthopedic Surgery: Protocol for a Systematic Review and Meta-analysis

**DOI:** 10.2196/28338

**Published:** 2022-04-15

**Authors:** Duanyang Wang, Pengbin Yin, Yi Li, Ming Chen, Xiang Cui, Shi Cheng, Yuan Lin, Jinglong Yan, Licheng Zhang, Peifu Tang

**Affiliations:** 1 Department of Orthopedics The Second Affiliated Hospital of Harbin Medical University Harbin Medical University Harbin China; 2 Department of Orthopedics Chinese PLA General Hospital Beijing China

**Keywords:** frailty, orthopedic surgery, systemic review, meta-analysis, older adults, elderly, surgery, orthopedics

## Abstract

**Background:**

Frailty is an aggregate expression of susceptibility to adverse health outcomes because of age- and disease-related deficits that accumulate across multiple domains. Previous studies have found the presence of preoperative frailty is associated with an increased risk of adverse outcomes. The number of older adults undergoing orthopedic surgery is rapidly increasing. However, there has been no evidence-based study on the relationship between frailty and outcomes in patients undergoing orthopedic surgery.

**Objective:**

The aims of this study are to investigate the association between frailty and outcomes in patients who underwent orthopedic surgery as well as patient factors associated with frailty.

**Methods:**

The methods to be used for this systematic review are reported according to the PRISMA-P (Preferred Reporting Items for Systematic Review and Meta-analysis Protocols) 2015 checklist. An extensive search will be conducted in PubMed, Embase, the Cochrane Library, and other mainstream databases. Any study where patients undergoing orthopedic surgery were assessed using a defined or validated measure of frailty and the association of frailty with patient factors and/or outcomes was reported will be included. A total of 2 researchers will independently screen articles for inclusion, with disagreements resolved by a third reviewer. We will perform a narrative synthesis of the factors associated with frailty, prevalence of frailty, effect of frailty on patient outcomes, and interventions for patients who are frail. A meta-analysis focusing on individual factors associated with frailty and the effect of frailty on patient outcomes will be performed, if applicable. The risk of bias will be evaluated. A subgroup analysis and sensitivity analysis will be performed.

**Results:**

Literature searches were conducted in September 2021 and the review is anticipated to be completed by the end of July 2022.

**Conclusions:**

This systematic review and meta-analysis will provide an overview of frailty and investigate the relationship between frailty and patient outcomes as well as the relationship between patient factors and frailty in patients undergoing orthopedic surgery. This study could potentially increase patients’ awareness of the outcomes associated with frailty, compel clinical specialties to further acknowledge the concept of frailty, and enhance the development of assessment instruments and tools for frailty.

**Trial Registration:**

PROSPERO CRD42020181846; https://www.crd.york.ac.uk/prospero/display_record.php?RecordID=181846

**International Registered Report Identifier (IRRID):**

DERR1-10.2196/28338

## Introduction

Frailty is characterized by a decline in function across multiple physiological systems, accompanied by an increased vulnerability to stressors [1-3]. It occurs in adults at any age but is more prevalent in older adults. Furthermore, 2 studies have separately shown that the weighted average estimate of frailty is 9.9% and 11%, and that frailty is more prevalent among women than men [4,5]. However, for individuals ages 85 years and older, the prevalence of frailty is 39.1% for men and 45.1% for women [6]. Due to the aging population, the prevalence of frailty is increasing and the condition is gaining global attention [5].

Evidence-based studies have confirmed the association between frailty and increased mortality, hospitalization, falls, and admission to long-term care among the general population [7]. Moreover, robust meta-analyses have concluded that patients with frailty who undergo surgery such as vascular [8,9], cardiac [10,11], and general surgery [12,13] are at a higher risk of short- and medium-term mortality and postoperative complications. Therefore, numerous clinical specialties have taken the concept of frailty into consideration. Assessment instruments and tools, such as the Frailty Index [14], have been developed and modified to be specific to the condition and to various specialties.

Frailty is always associated with sarcopenia. Sarcopenia, which can be assessed by cross-sectional imaging, is defined as a progressive and generalized skeletal muscle disorder and is also associated with increased adverse outcomes [15]. More and more studies about older adults are focusing on sarcopenia. Additionally, sarcopenia has been used as a proxy measure of frailty [16].

Patients undergoing orthopedic surgery represent a distinct population in terms of demographic parameters, comorbidities, and other factors. Although orthopedic surgeons are treating an aging population, there remains a lack of vigilance in identifying patients who are frail. Also, to the best of our knowledge, there is no robust evidence-based study on the relationship between frailty and outcomes in patients undergoing orthopedic surgery [4]. Therefore, this systematic review and meta-analysis aims to investigate the association between frailty and patient outcomes and to recognize factors associated with frailty in patients undergoing orthopedic surgery.

## Methods

### Protocol Registration

The methods of this systematic review are reported according to the PRISMA-P (Preferred Reporting Items for Systematic Review and Meta-analysis Protocols) 2015 checklist [17,18]. The review protocol was registered with PROSPERO (the International Prospective Register of Systematic Reviews; CRD42020181846). Any amendments to this protocol will be updated on PROSPERO and documented accordingly.

### Participants

This systematic review will include studies reporting on patients with symptomatic or suprathreshold (for treatment) orthopedic pathologies. Patients undergoing orthopedic procedures will be included for meta-analysis, if applicable. Orthopedic pathologies will include, but are not limited to, arthritis, bone fractures, intervertebral disc herniation, and sports injuries. Orthopedic procedures will include, but are not limited to, arthroplasty, amputation, internal fixation, spinal canal decompression, and spinal fusion. Included patients should have their frailty status measured using a defined or previously validated measure of frailty. Those whose frailty was only measured after intervention or who were defined as prefrail will be excluded.

### Exposure

Studies where frailty was measured by research-based assessment tools will comprise the “exposure group.”

### Controls

Studies where nonfrailty was measured by research-based assessment tools will be used as controls.

### Outcome Measures

This study will include 2 main outcome measures in accordance with our aims. The first is factors (such as demographic and social factors, clinical factors, lifestyle factors, and biological factors) associated with frailty in patients undergoing orthopedic surgery. The second is outcomes which include short- and long-term mortality, postoperative complications, and readmission.

### Search Strategy

The search strategies have been devised by the senior authors and accurately and repeatedly modified according to the analysis of the results after several tentative literature retrievals. A total of 2 independent reviewers will tentatively retrieve the studies in PubMed (MEDLINE) to ensure accurate retrieval. If their results are the same, an extensive search will be conducted in PubMed, Embase, the Cochrane Library, CINAHL, PsyclNFO, Scopus, and Web of Science. The literature search results will be saved and managed using EndNote X9 (Clarivate PLC). Detailed search strategies will be included in the first table of the paper reporting the study’s results.

### Inclusion Criteria

Studies will be included according to the following criteria:

1. Involving patients undergoing orthopedic surgery who were assessed using a defined or previously validated measure of frailty.
2. Reporting on the association of frailty with patient factors and/or outcomes.
3. Reporting on comparisons between frail and nonfrail patients undergoing orthopedic surgery as a separate subgroup.

### Exclusion Criteria

Studies will be excluded according to the following criteria:

1. Studies that only included patients undergoing orthopedic surgery who had asymptomatic disease, disease below the threshold for treatment, or disease treated with conservative management.
2. Studies where frailty was only measured post intervention or measured with continuous scores, without applying a defined “frailty threshold” to dichotomize the study population into frail and nonfrail groups.
3. Studies that did not report the methods used for measuring frailty.
4. Studies that did not include patients who were not frail.
5. Review articles, case reports, editorials or comments, or studies where the full text was not available.
6. Nonhuman studies

### Selection Process

The studies retrieved from the databases will be screened by 2 independent reviewers (PY and DW) according to their titles and abstracts. Studies will be marked as Y (yes), M (maybe), or N (no) by reviewers. If a study is marked as Y/Y or Y/M, it will advance to the next step of the review. If a study is marked as Y/N, M/M, or M/N, it will be considered a conflicted study and will be reviewed by the lead author and resolved through team discussion. If a study is marked as N/N, it will be excluded. The full texts of potentially eligible studies will be reviewed again by 2 independent reviewers (PY and DW) according to the inclusion and exclusion criteria.

Before the formal selection process, 100 studies will be chosen at random and 2 independent reviewers will perform an exercise according to the previously mentioned process. If sufficient agreement has been reached, the 2 independent reviewers will screen the full texts of the papers; otherwise, a second exercise will be performed until there is sufficient agreement between the reviewers.

### Quality Assessment

A quality assessment of the included studies will be performed by 2 independent reviewers (PY and DW). The Newcastle-Ottawa Scale (NOS) will be used to assess cohort studies and case-control studies [19]. An adapted version of the NOS will also be used to assess cross-sectional studies. In the subsequent meta-analysis, Version 2 of the Cochrane Risk of Bias tool and the ROBINS-E (Risk of Bias in Nonrandomized Studies of Exposures) tool will be used to assess the risk of bias of included studies where applicable.

### Quality of Evidence

The GRADE (Grading of Recommendations Assessment, Development, and Evaluation) system will be used to assess the quality of the evidence presented by the studies [20].

### Data Extraction

A data extraction form will be developed and piloted on no less than 5 included studies. The feedback from this will be used to guide the modification of the form. Then, 2 independent reviewers, (PY and DW), will abstract the data from included studies using the new version of the form. The main content of the extracted data will include basic information on the study, methodology for frailty measurement, patient frailty factors, assessment of quality, and outcomes. Any disagreements will be discussed and resolved with the lead reviewer. All extracted data will be stored in a Microsoft Excel (Microsoft Corp) spreadsheet. Detailed data extraction tools will be found in the second table of the paper publishing the results of this study.

### Data Synthesis

We will perform a narrative synthesis of the factors associated with frailty, prevalence of frailty in patients with various orthopedic pathologies, effect of frailty on the outcomes of the patients, and interventions to address frailty. After that, we will evaluate the homogeneity of the included studies. There may be an opportunity to conduct a meta-analysis focusing on individual factors associated with frailty and the effect of frailty on patient outcomes if 3 or more studies meet the requirements for the meta-analysis.

### Assessment of Heterogeneity

We will examine each included study to identify and assess the potential statistical, clinical, and methodological heterogeneity. The *P* value and I-squared statistic will be calculated to estimate the existence and magnitude of heterogeneity.

If the I-squared statistic is >50%, there would be extensive statistical heterogeneity. The judgement of clinical or methodological heterogeneity mainly depends on the authors’ clinical and methodological expertise. If the heterogeneity is strong, we will perform a systematic review in the place of a meta-analysis.

### Subgroup Analysis

To explore the source of the clinical or methodological heterogeneity, we will identify subgroups according to age, gender, frailty assessment tools, orthopedic diseases, and orthopedic operations. A narrative synthesis focusing on the subgroups will be performed. After that, meta-analyses will also be conducted for these same subgroups if we obtain sufficient data for the proposed groups.

### Sensitivity Analysis

Sensitivity analyses will be performed to test which study is the source of heterogeneity or whether the findings are robust. We will exclude low-quality studies if the studies selected have a wide range of quality. We will also perform a sensitivity analysis, restricting the meta-analysis to frequently used studies.

### Meta-regression

To further investigate the sources of clinical heterogeneity, a meta-regression will be performed if we obtain sufficient data. The metareg function in R (R Foundation for Statistical Computing) will be used to conduct the meta-regression with log-risk estimates. The standard error will be determined from 95% CIs for the log-risk estimates.

### Ethics Approval

Due to the nature of the study, there are no ethical concerns and informed consent will not be required.

## Results

The systematic review and meta-analysis are ongoing. The literature searches began in September 2021. Data abstraction and synthesis are expected to be completed at the end of April 2022. The review and analysis are anticipated to be finished by the end of July 2022. We plan to disseminate the results in a peer-reviewed journal.

## Discussion

### Necessity and Objective

Frailty is an emerging global health burden, with major implications for clinical practice and public health [4], The prevalence of frailty is expected to rise alongside population aging [5]. The identification of frailty is the first challenge facing health care providers. In the past decades, many frailty measurement instruments have been developed based on questionnaires, performance measures, electronic health record data, or a combination of these [4]. However, no consensus has been reached globally on how frailty should be measured [4]. Among prior research-based instruments, measurements based on electronic medical records seem to be more accepted and easily to be translated to clinical practice [21-23]. However, patients with different pathologies may have heterogeneous features and physicians in different specialties may evaluate their patients with their own focuses. Therefore, evidence-based research in the development and validation of frailty measurement tools for each specialty or even each pathology is required. The orthopedic specialist is increasingly challenged with treating older adults, a population with a higher prevalence of frailty and with multisystem disease and concomitant physical or cognitive impairments [24]. However, vigilance in frailty recognition and research on the association between frailty and patient outcomes is only recently beginning to emerge. Therefore, this systematic review will employ a rigorous methodology to summarize the existing data on patients undergoing orthopedic surgery who are frail. Our main objectives are to describe the characterization and measurement of frailty along with the associated outcomes, estimate the prevalence of frailty among patients undergoing orthopedic surgery, investigate patient factors associated with frailty in patients undergoing orthopedic surgery, and provide robust evidence on the associations of frailty with clinical outcomes.

### Outlook

We believe the results of this review will inform clinicians, patients, and health care providers of the best available evidence about the impact of frailty in patients undergoing orthopedic procedures. We also expect that our findings will fill certain gaps as well as trigger further research to enhance clinical decision-making with a focus on patient-important outcomes.

### Strengths

To the best of our knowledge, this review is anticipated to be the first to (1) describe and critique the tools which are used to assess the frailty in patients undergoing orthopedic surgery and the quality of evidence for their use, (2) investigate the association between frailty and patient outcomes, and (3) recognize patient factors associated with frailty in patients undergoing orthopedic surgery. The strength of our study lies in the nature of systematic review and meta-analysis study types, which adhere to established eligibility criteria, predefine outcomes, and incorporate multiple independent reviewers to minimize bias and increase reproducibility.

### Limitations

The conclusions of this study will be limited by the number and quality of studies included. There may be heterogeneity caused by differences in the study populations, interventions, and outcome measures.

### Conclusions

This systematic review and meta-analysis will provide an overview of frailty and investigate the relationship between frailty and outcomes and the relationship between patient factors and frailty in patients undergoing orthopedic surgery. This study could potentially increase patients’ awareness of the outcomes associated with frailty, compel clinical specialties to embrace the concept of frailty, and enhance the development of assessment instruments and tools.

## Abbreviations

GRADE: Grading of Recommendations Assessment, Development, and Evaluation
NOS: Newcastle-Ottawa Scale
PRISMA-P: Preferred Reporting Items for Systematic Review and Meta-analysis Protocols
PROSPERO: Prospective Register of Systematic Reviews
ROBINS-E: Risk of Bias in Nonrandomized Studies of Exposures
